# Supplementary material for: Identification of Anther Length QTL and Construction of Chromosome Segment Substitution Lines of Oryza longistaminata
Source: Plants (Basel). 2019 Sep 29;8(10):388. doi: 10.3390/plants8100388 (PMC6843423; doi:10.3390/plants8100388)
Supplement: Supplementary file 1 [file plants-08-00388-s001.zip › plants-595584-revised3-supplementary/supplementals/FigS1.pdf]

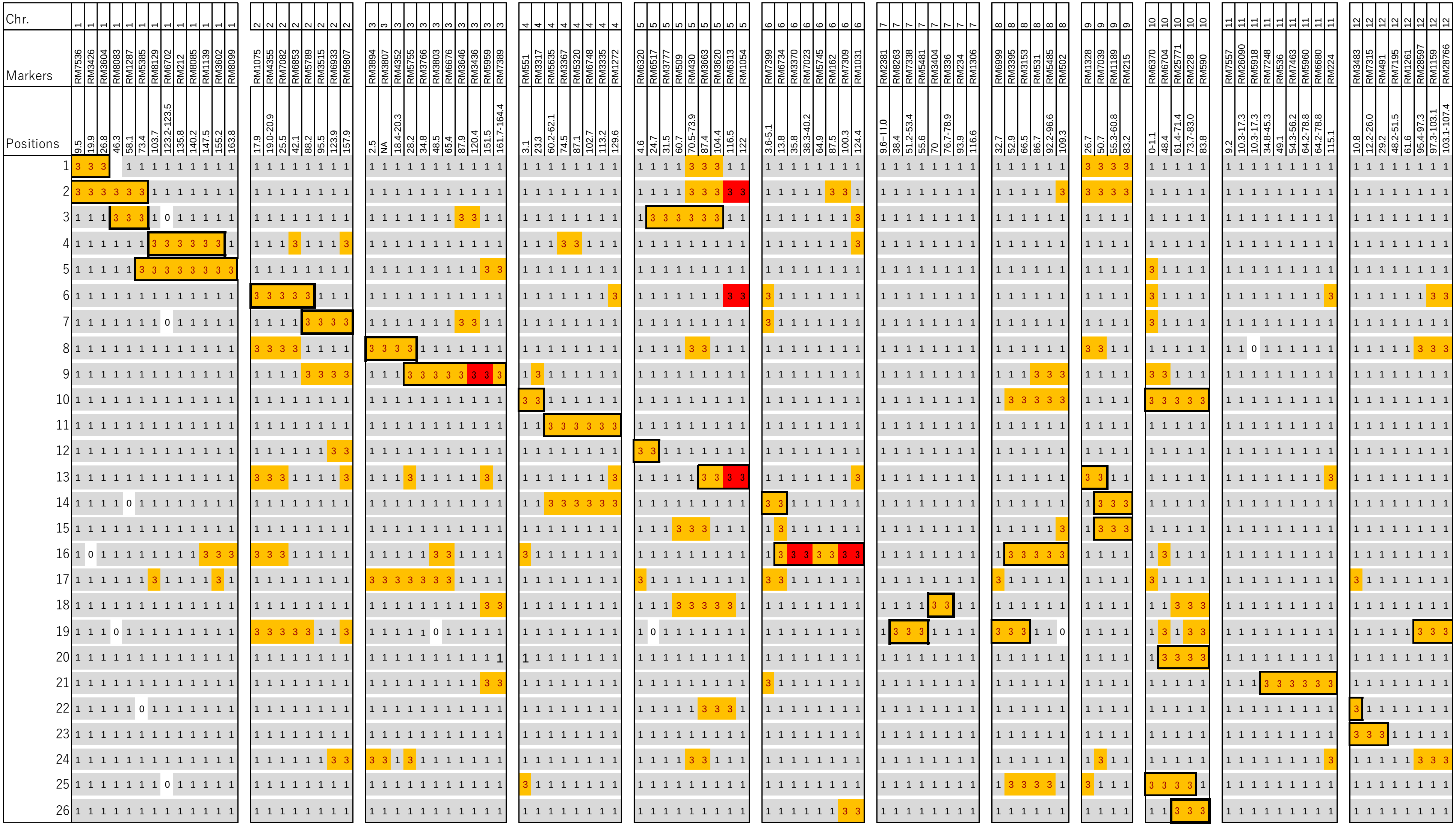


**Figure S1.** Graphical genotypes of $BC_3F_1$ for candidates for introgression lines of W1508 in T65 genetic background. Graphical genotypes: yellow, heterozygous; red, homozygous for the W1508 allele. Target regions for regional QTL analysis and target of fixation for homozygous genotypes for W1508 were enclosed by black boxes.
