# Supplementary material for: Identification of Anther Length QTL and Construction of Chromosome Segment Substitution Lines of Oryza longistaminata
Source: Plants (Basel). 2019 Sep 29;8(10):388. doi: 10.3390/plants8100388 (PMC6843423; doi:10.3390/plants8100388)
Supplement: Supplementary file 1 [file plants-08-00388-s001.zip › plants-595584-revised3-supplementary/supplementals/FigS2.pdf]

Model	Chr.	3	6	6	5	8	4	4 and 6	10	9	3 and 9	pLOD
	Position (cM)	136.5~138.5	25.6~33.6	99.6~105.6	118.6~120.6	49.6~63.6	75.1		1.1	72.7		
	Validated QTL	*qATL3*	*qATL6.1*	*qATL6.2*	*qATL5*							
	Effect	Main	Main	Main	Main	Main	Main	Interaction	Main	Main	Interaction	
1		1	0	0	0	0	0	0	0	0	0	1.75
2		1	1	0	0	0	0	0	0	0	0	4.22
3		1	1	1	0	0	0	0	0	0	0	5.35
4		1	1	1	1	0	0	0	0	0	0	8.05
5		1	1	1	1	1	0	0	0	0	0	7.96
6		1	1	1	1	1	1	0	0	0	0	7.90
7		1	1	1	1	1	1	1	0	0	0	7.62
8		1	1	1	1	1	1	1	1	0	0	7.03
9		1	1	1	1	1	1	1	1	1	1	6.40
10		1	1	1	1	1	1	1	1	0	0	7.03
11		1	1	1	1	1	1	1	0	0	0	7.73
12		1	1	1	1	1	1	0	0	0	0	7.90
13		1	1	1	1	1	0	0	0	0	0	7.96
14		1	1	1	1	0	0	0	0	0	0	8.05
15		1	0	1	1	0	0	0	0	0	0	5.61
16		0	0	1	1	0	0	0	0	0	0	3.37
17		0	1	0	0	0	0	0	0	0	0	1.55

Figure S2. Model selections in multiple QTL mapping including main and interaction effects for anther length.
0 and 1 represent excluding and including QTL to models, respectively. Model 4 and 14 showed the highest penalty LOD score, suggesting that the model including *qQTL3*, *qATL5*, *qATL6.1*, and *qATL6.2* was the best model explaining anther length in the BC_3F_1 population.
